# Outcomes of Ceftriaxone Compared With Cefazolin or Nafcillin/Oxacillin for Outpatient Therapy for Methicillin-Sensitive *Staphylococcus aureus* Bloodstream Infections: Results From a Large United States Claims Database

**DOI:** 10.1093/ofid/ofad662

**Published:** 2024-01-12

**Authors:** Yasir Hamad, Katelin B Nickel, Margaret A Olsen, Ige A George

**Affiliations:** Critical Care Medicine Department, National Institutes of Health, Bethesda, Maryland, USA; Division of Infectious Diseases, Department of Medicine, Washington University School of Medicine in St Louis, St Louis, Missouri, USA; Division of Infectious Diseases, Department of Medicine, Washington University School of Medicine in St Louis, St Louis, Missouri, USA; Division of Public Health Sciences, Department of Surgery, Washington University School of Medicine in St Louis, St Louis, Missouri, USA; Division of Infectious Diseases, Department of Medicine, Washington University School of Medicine in St Louis, St Louis, Missouri, USA

**Keywords:** bloodstream infection, ceftriaxone, methicillin-sensitive *Staphylococcus aureus*, MSSA, outpatient parenteral antibiotic therapy

## Abstract

**Background:**

Ceftriaxone is a convenient option for methicillin-sensitive *Staphylococcus aureus* (MSSA) outpatient parenteral antimicrobial therapy (OPAT), but population-based studies for its effectiveness are lacking.

**Methods:**

In this retrospective cohort, a large insurance claims database was queried from 2010 to 2018 for adults with MSSA bloodstream infection (BSI). Patients discharged on OPAT on cefazolin or oxacillin/nafcillin were compared with ceftriaxone with respect to 90-day hospital readmission with the same infection category and 90-day all-cause readmission using logistic regression models.

**Results:**

Of 1895 patients with MSSA BSI, 1435 (75.7%) patients received cefazolin, oxacillin, or nafcillin and 460 (24.3%) ceftriaxone. Readmission due to the same infection category occurred in 366 (19.3%), and all-cause readmission occurred in 535 (28.3%) within 90 days. Risk factors significantly associated with readmission with the same infection category were the oldest sampled age group (61–64 years: adjusted odds ratio [aOR], 1.47 [95% confidence interval {CI}, 1.01–2.14]), intensive care unit stay during index admission (aOR, 2.33 [95% CI, 1.81–3.01]), prosthetic joint infection (aOR, 1.96 [95% CI, 1.18–2.23]), central line–associated BSI (aOR, 1.72 [95% CI, 1.33–2.94]), and endocarditis (aOR, 1.63 [95% CI, 1.18–2.23]). Ceftriaxone was not associated with increased risk of readmission with the same infection category (aOR, 0.89 [95% CI, .67–1.18]), or 90-day all-cause readmission (aOR, 0.86 [95% CI, .66–1.10]) when compared with oxacillin/nafcillin/cefazolin.

**Conclusions:**

In this cohort of MSSA BSI patients discharged on OPAT, there were no differences in outcomes of readmission with the same infection and 90-day all-cause readmission in patients treated with ceftriaxone compared to oxacillin/nafcillin or cefazolin. Patients with complicated BSIs such as endocarditis and epidural abscess were more likely to be prescribed cefazolin or oxacillin/nafcillin.

Methicillin-susceptible *Staphylococcus aureus* (MSSA) continues to be an important cause of both community-onset and hospital-acquired bloodstream infections (BSIs), with 90-day mortality rates of 20%–25% [1, 2]. Due to the risk of recurrence and metastatic septic complications, the Infectious Diseases Society of America guidelines and expert opinion recommend prolonged parenteral therapy for *S aureus* BSIs [1]. Expert opinion recommends treatment with a β-lactamase–resistant penicillin (nafcillin or oxacillin) or cefazolin to treat MSSA BSIs; however, the optimal choice of therapy is unclear [1, 3, 4]. An alternative treatment option is ceftriaxone, which has a US Food and Drug Administration–labeled indication for MSSA septicemia with a favorable long-term side effect profile and is administered once daily, making it convenient for outpatient parenteral antimicrobial therapy (OPAT) [5]. However, clinicians may be hesitant to use ceftriaxone for MSSA infections, particularly in serious infections like BSI, due to lack of consistent evidence for effectiveness in the literature [6].

Pharmacokinetic and pharmacodynamic studies on the use of ceftriaxone for MSSA infection are concerning for inadequate bactericidal activity with once-daily dosing regimens when compared to other antistaphylococcal β-lactams [7, 8]. However, these studies were conducted to simulate an active infection with high burden of disease, and whether the results apply to more stable patients being discharged on OPAT is unclear.

Few retrospective studies have evaluated ceftriaxone for the treatment of MSSA BSIs; however, these studies have conflicting results with limited sample sizes [9–11]. We recently conducted a retrospective review of 243 patients with MSSA BSI discharged on OPAT from a single academic center and found that ceftriaxone was increasingly used to treat MSSA infections [12]. There were no significant differences in microbiological failure, 90-day all-cause mortality, or readmission due to MSSA BSI in patients treated with ceftriaxone compared to those treated with oxacillin or cefazolin therapy [12]. A recent meta-analysis included 12 retrospective studies evaluating the safety and effectiveness of ceftriaxone in MSSA BSIs found that use of ceftriaxone was not different from oxacillin and cefazolin in clinical and microbiological cure and in 30- and 90-day readmission rates and mortality [13].

There are no population-based studies on the use of ceftriaxone for MSSA BSIs that compare outcomes for MSSA BSI to standard β-lactam antibiotics like oxacillin/nafcillin or cefazolin for OPAT. We used a large commercial health insurance claims database to assess factors associated with ceftriaxone OPAT use in MSSA BSI and to study outcomes in patients discharged on ceftriaxone compared with cefazolin or nafcillin/oxacillin.

## METHODS

### Design

This is a retrospective cohort study of MSSA BSI patients discharged on OPAT. The use of ceftriaxone was compared with cefazolin or nafcillin/oxacillin. We followed guidance provided by Strengthening the Reporting of Observational Studies in Epidemiology (STROBE) initiative.

### Data

Data were obtained from the Merative MarketScan Commercial Database, an administrative claims database that contains medical claims from inpatient and outpatient encounters as well as outpatient prescription-drug claims for >150 million enrolled persons and their dependents covered under a variety of employer-sponsored and other health plans. All adult patients aged 18–64 years with an index hospitalization coded with an *International Classification of Diseases, Ninth* or *Tenth Revision, Clinical Modification* (*ICD-9/10-CM*) diagnosis code for MSSA septicemia (038.11, A41.01) from 1 January 2010 to 30 September 2018 were identified. Patients with MSSA BSI who received a prescription or *Healthcare Common Procedure Coding System* (HCPCS) code for injectable solutions of cefazolin, ceftriaxone, or oxacillin/nafcillin plus *Current Procedural Terminology* (CPT) and/or HCPCS codes for home or outpatient infusion therapy administration and/or supplies within 7 days after hospital discharge were considered to have received OPAT [14]. Patients were required to have medical and prescription coverage for 1 year prior to the index hospitalization to capture baseline comorbidities.

Patients aged >64 years and those with baseline end-stage renal disease were excluded due to potentially incomplete claims data due to enrollment in the Medicare program. Patients discharged from their index MSSA BSI hospitalization to a skilled nursing facility were excluded due to the inability to identify OPAT administered in the facility. Patients who received other antibiotics with antistaphylococcal activity, including daptomycin, clindamycin, vancomycin, linezolid, telavancin, and ceftaroline within 7 days of index hospital discharge were also excluded. Rifampin and gentamicin used as adjunctive agents during OPAT were captured. If a patient had >1 hospitalization associated with OPAT during the study time that met all inclusion criteria, only the first hospitalization was included as an outcome.

### Variables

Demographic variables included were age, sex, and patient residence (rural vs urban, with urban defined as living in a metropolitan statistical area). Comorbidities were defined using *ICD-9/10-CM* diagnosis codes in the index hospitalization and the year prior to the index admission, using primarily the Elixhauser classification, requiring coding in ≥1 inpatient facility hospitalization (including the index admission) and/or ≥2 provider or outpatient facility (excluding diagnostic) claims spaced at least 30 days apart [15, 16]. *ICD-9/10-CM* diagnosis codes were used to identify infectious diagnoses during the index hospitalization; an admission could be associated with >1 infectious diagnosis. Infections were categorized into clinically relevant categories (eg, central line–associated bacteremia [CLABSI], bone and joint infection, skin and soft tissue infection, surgical site infection, endocarditis, or pneumonia) (Supplementary Table 1) [17]. Evaluation by an infectious diseases (ID) physician during the index hospitalization, CLABSI, cardiovascular implantable electronic device, and valve replacement surgery done in the year prior and during the index admission were captured (Supplementary Table 1). Duration of OPAT was calculated using periods of continuous home infusion therapy (HIT), continuous antibiotics of the same name, then taking overlapping HIT and antibiotic periods (allowing 5-day gaps of both to consider continuous) to create a period of OPAT starting with the last start date of HIT or antibiotics and ending at the first ending date of HIT or antibiotics.

### Outcomes

Outcomes were readmission to a hospital with same infectious category as the index admission within 90 days after index hospital discharge, and all-cause readmission within 90 days. The former was chosen with the pragmatic aim to identify patients who developed treatment failure or relapse of infection with any of the different infection categories as identified in the index hospitalization (eg, if a patient was admitted with endocarditis and prosthetic joint infection [PJI] and was readmitted with either endocarditis or PJI, this would be identified as an outcome). Planned short readmissions for chemotherapy were excluded as an outcome.

### Statistical Analyses

Descriptive statistics were used for the demographic and clinical characteristics of the study population. Factors associated with the use of ceftriaxone and readmission outcomes were analyzed by using χ^2^ tests with binary and categorical variables, while continuous variables were analyzed using Student *t* test and Mann-Whitney *U* test, as appropriate.

We assessed potential risk factors for readmission using univariate and multivariable logistic regression models. For the multivariable model, variables were specified by selecting clinically meaningful factors that could potentially be associated with readmission, which included type of infection (eg, endocarditis) and choice of antibiotic therapy as well as statistically significant variables on univariate analysis. Nonsignificant variables in the multivariable regression were removed via backward elimination until the final listed model was achieved. A subgroup analysis was performed of patients with endocarditis, as these patients have associated higher morbidity and mortality.

We developed a propensity score for treatment with ceftriaxone by balancing potential confounders along with variables related to the outcomes across the 2 treatment groups in a nonparsimonious approach. The propensity score (PS) was inversely weighted for ceftriaxone (1 / PS) and oxacillin/nafcillin/cefazolin (1 /[1 – PS]), respectively [18, 19]. The variables included were demographic and comorbidities, intensive care unit (ICU) stay, and infectious categories including endocarditis. We assessed the balance of observed variables between the 2 treatment groups; absolute standardized mean differences of <0.1 in the weighted population were considered adequate (Supplementary Table 3). We constructed logistic regression models for the outcomes (90-day readmission with the same infection category), including the treatment group and weighting by the inverse probability of treatment. All analyses were performed using SAS version 9.4 software (SAS Institute, Cary, North Carolina), and for all tests *P* < .05 was considered significant.

## RESULTS

### Descriptive Statistics

During the study period from January 2010 until September 2018, 1895 patients were coded for MSSA septicemia and were administered OPAT with oxacillin/nafcillin/cefazolin, or ceftriaxone treatment within 7 days of hospital discharge (Supplementary Figure 1). The median age of patients was 54 years, and 63% were male. The most common comorbidities in the cohort were hypertension (50%), diabetes mellitus (33%), obesity (25%), and valvular heart disease (11%). Fifty percent of patients were managed in an ICU during the index hospitalization. Sixty-nine percent of patients received an ID consultation during the index MSSA BSI admission (Table 1). Skin and soft tissue infection was the most common infection category associated with MSSA BSI diagnosis, occurring in 757 (40%). Other infections identified during the index MSSA BSI hospitalization included surgical site infection (558 [30%]), pneumonia (356 [19%]), and osteomyelitis (335 [18%]). The majority of patients (69%) had an echocardiogram during the index admission, and 276 (15%) patients had endocarditis. Twenty-nine (11%) patients with endocarditis had valve replacement during the index hospitalization. The median index hospital stay was 7 days (interquartile range [IQR], 5–12 days).

**Table 1.** Clinical Characteristics of Patients With Methicillin-Susceptible *Staphylococcus aureus* Bloodstream Infection

Variable	Total (N = 1895)	Oxacillin/Nafcillin/Cefazolin (n = 1435)	Ceftriaxone (n = 460)	*P* Value
Demographic characteristics				
Age, y, median (IQR)	54 (45–60)	50.7 (45–60)	50.9 (46–60)	.157
Sex (male)	1192 (62.9)	903 (62.9)	289 (62.8)	.964
Residing in an urban area	1615 (87.7)	1229 (88.1)	386 (86.1)	.282
Comorbidities				
Congestive heart failure	195 (10.3)	154 (10.7)	41 (8.9)	.290
Diabetes	630 (33.3)	470 (32.7)	160 (34.8)	.432
Chronic kidney disease	132 (7.0)	97 (6.7)	35 (7.6)	.528
Hypertension	947 (50.0)	713 (49.6)	234 (50.8)	.668
Solid tumors	136 (7.2)	36 (6.9)	31 (6.7)	.546
Hematological malignancies	58 (3.1)	45 (3.1)	13 (2.8)	.871
Valvular heart disease	204 (10.8)	166 (11.6)	38 (8.2)	.047
Metastatic cancer	136 (7.2)	105 (7.3)	31 (6.7)	.755
Pulmonary circulation disease	142 (7.5)	116 (8.1)	26 (5.6)	.102
Peripheral vascular disease	128 (6.8)	99 (6.9)	29 (6.3)	.748
Obesity	482 (25.4)	373 (25.9)	109 (23.7)	.356
Drug abuse	104 (5.5)	81 (5.6)	23 (5)	.639
CIED implantation during the past year	89 (4.7)	70 (4.8)	19 (4.1)	.509
Valve replaced during the past year	13 (0.7)	9 (0.63)	4 (0.87)	.583
Index admission characteristics				
Length of index hospital stay, d, median (IQR)	7 (5–12)	8 (6–12)	7 (5–10)	<.001
ICU stay	957 (50.0)	727 (50.6)	230 (50)	.804
ID consultation	1308 (69.0)	1013 (70.5)	295 (64.2)	.009
Echocardiography done	1306 (68.9)	1036 (72.2)	270 (54.6)	<.001
Hospitalization in the 30 d prior to the index admission	356 (18.8)	281 (19.5)	75 (16.5)	.117
Valve replaced during index admission	29 (1.5)	23 (1.6)	6 (1.3)	.650
Type of infection at index admit				
Osteomyelitis	335 (17.7)	260 (18.2)	75 (16.3)	.375
Septic arthritis	251 (13.3)	198 (13.8)	53 (11.5)	.210
Prosthetic joint infection	162 (8.6)	129 (6.8)	33 (7.1)	.220
Central line–associated bacteremia	175 (9.2)	137 (7.2)	38 (8.2)	.407
Infection of vascular device	192 (10.1)	156 (10.8)	36 (7.8)	.596
Skin and soft tissue infection	757 (40.0)	587 (40.1)	170 (36.9)	.132
Surgical site infection	558 (29.5)	437 (30.4)	121 (26.3)	.099
Epidural abscess	200 (10.6)	170 (11.9)	30 (6.5)	.002
Endocarditis	276 (14.6)	229 (15.9)	47 (10.2)	.002
Pneumonia	356 (18.8)	255 (17.7)	101 (22)	.045
OPAT characteristics				
OPAT duration, d, median (IQR)	15 (7–28)	15 (7–29)	15 (8–29)	.076
Home-based OPAT	1712 (90.3)	1351 (94.1)	361 (78.5)	<.001

Data are presented as No. (%) unless otherwise indicated.

Abbreviations: CIED, cardiac implantable electronic devices; ID, infectious diseases; IQR, interquartile range; OPAT, outpatient parenteral antimicrobial therapy.

A total of 1435 patients (76%) received either oxacillin/nafcillin/cefazolin, while 460 (24%) received ceftriaxone as OPAT. The median duration of OPAT was 15 days (IQR, 7–28 days). Adjunctive rifampin and or gentamicin was provided in only 18 patients (<1%). Oxacillin/nafcillin/cefazolin was more commonly prescribed for OPAT compared with ceftriaxone for patients who had a diagnosis of endocarditis (229/1435 [16%] vs 47/460 [10%]; *P* < .01) and epidural abscess (170/1432 [12%] vs 30/460 [7%]; *P* < .01). Patients with pneumonia were more likely to receive ceftriaxone than oxacillin/nafcillin/cefazolin (101/460 [22%] vs 255/1435 [18%]; *P* < .05). There was no difference in the proportion of patients receiving oxacillin/nafcillin/cefazolin versus ceftriaxone who were admitted to the ICU (51% vs 50%; *P* = .8) or those who had a diagnosis of sepsis/septicemia (42% vs 46%; *P* = .22) (Table 1).

### Outcomes Analysis

Readmission with any of the same infection category occurred in 366 (19%) patients within 90 days of index hospital discharge and did not vary by OPAT antibiotic. Seventy-eight of 460 (17%) patients were readmitted with the same infection category in the ceftriaxone group compared to 288 of 1435 (20%) in the oxacillin/nafcillin/cefazolin group (adjusted odds ratio [aOR], 0.89 [95% confidence interval {CI}, .67–1.18]) (Table 2 and Supplementary Table 2). Risk factors significantly associated with readmission due to the same infection category in multivariable analysis were age 61–64 years (aOR, 1.47 [95% CI, 1.01–2.14]), obesity (aOR, 1.40 [95% CI, 1.07–1.82]), ICU stay during index admission (aOR, 2.33 [95% CI, 1.81–3.01]), hospitalization within the 30 days prior to index admission (aOR, 1.59 [95% CI, 1.20–2.01]), PJI (aOR, 1.96 [95% CI, 1.18–2.23]), CLABSI (aOR, 1.72 [95% CI, 1.33–2.94]), and endocarditis (aOR, 1.63 [95% CI, 1.18–2.23]) (Table 2). Similarly, there was no difference in the rates of all-cause readmissions (114/460 [25%] in the ceftriaxone group versus 421/1435 [29%] in the oxacillin/nafcillin/cefazolin group; OR, 0.79 [95% CI, .62–1.03]). Risk factors significantly associated with all-cause readmissions were similar: age 61–64 years (aOR, 1.46 [95% CI, 1.04–2.06]), ICU stay (aOR, 2.42 [95% CI, 1.92–3.02]), hospitalization within 30 days (aOR, 1.73 [95% CI, 1.37–2.28]), PJI (aOR, 1.74 [95% CI, 1.19–2.53]), CLABSI (aOR, 2.48 [95% CI, 1.76–3.49]), and endocarditis (aOR, 1.60 [95% CI, 1.22–2.14]) (Table 2).

**Table 2.** Factors Associated With Readmission in Multivariable Analysis (N = 1895)

Variable	Readmission With the Same Infection Category				All Readmissions			
	Yes (n = 366 [19.3])	No (n = 1529 [80.7])	*P* Value	aOR (95% CI)	Yes (n = 535 [28.3])	No (n = 1360 [71.7])	*P* Value	aOR (95% CI)
Age, y								
18–40	71 (19.4)	261 (17.1)	.407	1.38 (.92–2.06)	102 (19.1)	230 (16.7)	.549	1.26 (.90–1.77)
41–50	60 (16.4)	319 (20.9)	Ref	Ref	93 (35.3)	289 (21.3)		Ref
51–60	132 (36.1)	575 (37.6)	.733	1.21 (.85–1.76)	189 (35.3)	518 (38.1)	.268	1.05 (.79–1.46)
61–64	103 (28.1)	374 (24.5)	.036	1.47 (1.01–2.14)	151 (28.2)	326 (28.2)	.003	1.46 (1.04–2.06)
Comorbidities								
Obesity	113 (30.9)	369 (24.1)	.011	1.40 (1.07–1.82)	153 (28.6)	329 (24.1)	.052	1.23 (.99–1.61)
ICU stay	245 (66.9)	712 (46.6)	<.001	2.33 (1.81–3.01)	355 (66.4)	602 (44.2)	<.001	2.42 (1.92–3.02)
Hospitalization in the 30 d prior to the index admission	99 (27.1)	257 (16.8)	.001	1.59 (1.20–2.01)	145 (27.1)	211 (15.5)	<.001	1.73 (1.37–2.28)
Central line–associated bacteremia	48 (13.1)	127 (8.3)	.001	1.72 (1.33–2.94)	76 (14.2)	99 (7.2)	<.001	2.48 (1.76–3.49)
Prosthetic joint infection	43 (11.8)	119 (7.8)	.001	1.96 (1.25–2.23)	56 (10.4)	106 (7.8)	.003	1.74 (1.19–2.53)
Endocarditis	71 (19.4)	205 (13.4)	.002	1.63 (1.18–2.23)	100 (18.6)	176 (12.9)	.001	1.60 (1.22–2.14)
OPAT antibiotic								
Oxacillin/nafcillin/cefazolin	288 (78.6)	1147 (75)	Ref	…	421 (78.7)	1014 (74.6)	Ref	…
Ceftriaxone	78 (21.3)	382 (25.0)	.428	0.89 (.67–1.18)	114 (21.3)	346 (25.4)	.230	.86 (.66–1.10)

Data are presented as No. (%) unless otherwise indicated.

Abbreviations: aOR, adjusted odds ratio; CI, confidence interval; ICU, intensive care unit; OPAT, outpatient parenteral antimicrobial therapy.

The effect of treatment group assignment on outcomes was also analyzed using inverse weighting by the PS for receipt of ceftriaxone OPAT. There was no difference in the outcomes of readmission with the same infection category based on antibiotic choice (aOR, 1.01 [95% CI, .86–1.19]) and all-cause readmission (aOR, 0.91 [95% CI, .78–1.05]) (Table 3).

**Table 3.** Antibiotic Choice and Risk of Readmission, With Propensity Score Inverse Weighting: Logistic Regression Model (N = 1895)

Variable	Readmission With the Same Infection Category				All Readmissions			
	Yes (n = 366 [19.3])	No (n = 1529 [80.7])	*P* Value	aOR (95% CI)	Yes (n = 535 [28.3])	No (n = 1360 [71.7])	*P* Value	aOR (95% CI)
Oxacillin/nafcillin/cefazolin	288 (78.6)	1147 (75)	Ref	…	421 (78.7)	1014 (74.6)	Ref	…
Ceftriaxone adjusted (PS-weighted model)	78 (21.3)	382 (25.0)	.937	1.01 (.86–1.19)	114 (21.3)	346 (25.4)	.203	0.91 (.78–1.05)

Data are presented as No. (%) unless otherwise indicated.

Abbreviations: aOR, adjusted odds ratio; CI, confidence interval; PS, propensity score.

### Subanalysis of Endocarditis

Among the subset of patients with endocarditis during the index admission, the majority were treated with oxacillin/nafcillin/cefazolin (229/276 [83%]). There was no difference in the outcomes of readmission with the same infection category in univariate analysis among those with endocarditis during the index admission based on type of OPAT: 9 (19%) in the ceftriaxone group versus 62 (27%) in the oxacillin/nafcillin/cefazolin group, respectively (*P* = .257, Supplementary Table 4). There was also no difference in all-cause readmissions in the endocarditis subgroup based on the type of antibiotics used for OPAT in univariate analysis (15 [32%] in the ceftriaxone group versus 85 [37%] in the oxacillin/nafcillin/cefazolin group, respectively; *P* = .499).

## DISCUSSION

This large observational study of nonelderly commercially insured patients from across the United States showed similar risk of readmission due to the same infection category and all-cause readmission for patients treated with ceftriaxone OPAT compared to nafcillin/oxacillin or cefazolin OPAT for MSSA BSI. Ceftriaxone provides advantages to usual antibiotics (nafcillin/oxacillin and cefazolin) for treatment of MSSA BSI, with once-daily dosing and a short infusion time [20]. A survey of OPAT patients at our institution revealed that less frequent administration of antibiotics (once or twice daily) was associated with significantly better adherence when compared with more frequent dosing regimens. Eighty-three percent of patients with poor adherence were prescribed more frequent intravenous antibiotic dosing compared to only 24% of those without a missed dose, arguing that ceftriaxone as compared with more frequent dosing regimens of oxacillin or cefazolin would have better adherence and acceptability [21]. Historically, the use of ceftriaxone for invasive MSSA infection treatment is uncommon, with inconsistent data supporting the clinical utility of ceftriaxone for MSSA BSI [9, 10, 12]. The use of ceftriaxone in one-fourth of patients as seen in this study indicates that clinicians are using ceftriaxone more frequently now for the management of MSSA BSI, which is likely driven by the convenience of an outpatient once-daily regimen of ceftriaxone.

In this study, ceftriaxone, when compared with nafcillin/oxacillin/cefazolin, showed no significant differences in the endpoints of all-cause readmission (aOR, 0.86 [95% CI, .66–1.10]) and readmission due to the same infection category as index admission (aOR, 0.89 [95% CI, .67–1.18]) in the multivariable model, with similar results in the PS-weighted model (Tables 2 and 3). This is consistent with findings from a recent meta-analysis that showed no significant difference in readmission rates among MSSA BSI patients discharged on ceftriaxone [13]. This research contributes to the existing body of knowledge by highlighting that in patients with MSSA BSI who are discharged on OPAT, the use of ceftriaxone presents a reasonable choice, emphasizing the need for a randomized clinical trial to substantiate these results.

Oxacillin/nafcillin/cefazolin was more commonly prescribed for OPAT for patients who had a diagnosis of endocarditis compared to ceftriaxone. This likely represents ID physicians’ preference to use oxacillin/cefazolin for endocarditis and complicated bacteremias in order to adhere to guideline recommendations [4]. Patients with endocarditis had higher risk of readmission with the same infection category (aOR, 1.63 [95% CI, 1.18–2.23]) and all-cause readmission (aOR, 1.60 [95% CI, 1.22–2.14]), compared to those with MSSA BSI without endocarditis, and likely reflects a group that is expected to have higher mortality. Although there were no differences in the readmission rates between the 2 antibiotic groups among the subgroup of patients with endocarditis in univariate analysis, similar to prior observational studies, the subset of patients with endocarditis treated with ceftriaxone was small (n = 47), resulting in low statistical power. Hence, we are unable to generalize the results of effectiveness of ceftriaxone to patients with endocarditis.

There are several limitations to this observational study. We used commercial claims data of adult patients aged <65 years, and our findings may not be generalizable to Medicare, Medicaid, uninsured, or older patient populations. We did not study patients who completed their treatment after readmission or who were discharged to nursing homes or skilled nursing facilities, and hence may have selected a relatively healthier population. However, the study included a wide variety of underlying infections including complicated infections such as endocarditis and epidural abscess.

We relied on insurance claims and were unable to review medical records, and hence were not able to determine the adequacy of source control measures, time to blood culture clearance, or duration or type of antibiotics administered during the index MSSA BSI inpatient stay. There could be issues with confounding by indication and bias of preferring certain antibiotics for particular diagnoses. We selected a cohort of patients who received nafcillin/oxacillin, cefazolin, or ceftriaxone throughout their OPAT course, and hence were unable to extrapolate this data to other antibiotics with MSSA activity. We used a conservative approach to define OPAT, requiring both a HIT CPT/HCPCS code indicating HIT and concomitant code for antimicrobial use or retail pharmacy fill for an intravenous antimicrobial, and this could have resulted in an underestimation of the number of patients prescribed OPAT. We did not attempt to delineate a primary diagnosis based on the positioning of the *ICD-9/10-CM* codes or based on diagnosis. A more serious infection (eg, endocarditis) would weigh more in the clinical decision on treatment duration or choice of antibiotics, and we felt that additional presence of less serious infection (eg, cellulitis) is less likely to bias the results.

Last, we could not assess mortality as a clinical endpoint, since it was not available in the MarketScan data for the entire study period, or the risk of emergence of antimicrobial-resistant organisms with ceftriaxone use. Readmissions could also have been due to treatment-related adverse effects, which was not specifically evaluated but could have been captured with the all-cause outcome definition. Despite these limitations, this is the largest study to date comparing ceftriaxone with other antibiotics of interest in OPAT for MSSA BSI, and provides a pragmatic comparison of concurrent treatment practices in OPAT for MSSA BSI.

## CONCLUSIONS

There was no difference in risk of readmission with the same infection category nor all-cause readmission with respect to use of oxacillin/nafcillin/cefazolin versus ceftriaxone for OPAT for MSSA BSI. Patients with complicated BSI such as endocarditis and epidural abscess were more likely to be prescribed cefazolin or oxacillin/nafcillin. With a potential once-daily dosing option, ceftriaxone might prove to be a viable alternative to cefazolin and oxacillin/nafcillin for patients with MSSA BSI discharged on OPAT. However, given the limitations, these results need to be validated in a randomized clinical trial.

## Supplementary Material

ofad662_Supplementary_Data

## Supplementary Data

Supplementary materials are available at *Open Forum Infectious Diseases* online. Consisting of data provided by the authors to benefit the reader, the posted materials are not copyedited and are the sole responsibility of the authors, so questions or comments should be addressed to the corresponding author.

## Notes

***Patient consent*.** This study was considered exempt by the Washington University School of Medicine Human Research Protection Office, as we used a limited data set that prohibits reidentification.

***Financial support*.** This project was supported by the Washington University Institute of Clinical and Translational Sciences which is, in part, supported by the National Institutes of Health/National Center for Advancing Translational Sciences (Clinical and Translational Science Award number UL1 TR002345).
