# Supplementary material for: Outcomes of Ceftriaxone Compared With Cefazolin or Nafcillin/Oxacillin for Outpatient Therapy for Methicillin-Sensitive Staphylococcus aureus Bloodstream Infections: Results From a Large United States Claims Database
Source: Open Forum Infect Dis. 2024 Jan 12;11(2):ofad662. doi: 10.1093/ofid/ofad662 (PMC10863560; doi:10.1093/ofid/ofad662)
Supplement: ofad662_Supplementary_Data [file ofad662_supplementary_data.docx]

**Supplementary Figure 1: Flow chart of included patients.**

**
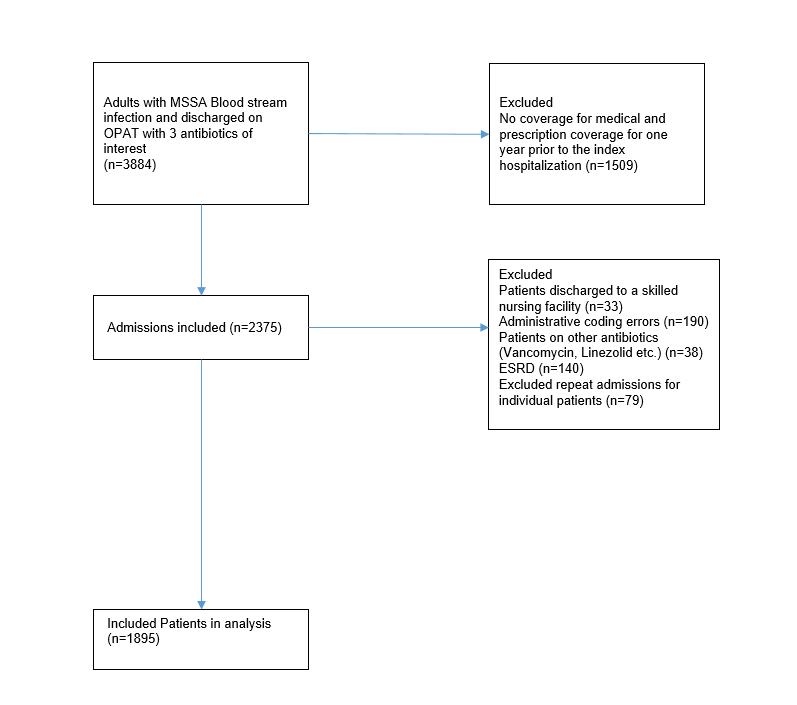
**

**Supplementary Table 1: Categories of infections along with diagnosis /procedure codes.**

| **DESCRIPTION OF CONDITION, PROCEDURE, OR DRUG** | **ICD-9-CM/ICD-10-CM DIAGNOSIS CODES** | **CPT-4/HCPCS, ICD-9-CM, OR ICD-10-PCS**  **PROCEDURE CODES** |
| --- | --- | --- |
| MSSA septicemia (required for study inclusion) | 038.11  A41.01 |  |
| HIT OPAT procedure code for OPAT (required for study inclusion). |  | **HCPCS codes for supplies**:  E0791, E0781, E0780, E0779, E0776, K0455, S1015, A4305, A4306  **HCPCS codes for HIT:**  S9494, S9497, S9500, S9501, S9502, S9503, S9504  **HCSPCS codes for HIT catheter care/insertion:**  S5501 , S5502, S5517, S5518, S5520, S5521, S5522, S5523, S5497, S5498  **CPT codes for Home infusion therapy** : 99601, 99602  **CPT Codes for infusion therapy:** 96365, 96366, 96367, 96368, 96374, 96375, 96376, 96379 |
| Endocarditis  (also including prosthetic valve endocarditis) | 421.0, 421.9  I33.0, I33.9 |  |
| Epidural Abscess  Brain abscess | 324.0, 324.1, 324.9  G06.0, G06.1, G06.2 |  |
| Osteomyelitis | 730.0x, 730.2x  M86.00, M86.10, M86.20, M86.011, M86.012, M86.019, M86.111, M86.112, M86.119, M86.211, M86.212, M86.219, M86.021, M86.022, M86.029, M86.121, M86.122, M86.129, M86.221, M86.222, M86.229, M86.031, M86.032, M86.039, M86.131, M86.132, M86.139, M86.231, M86.232, M86.239, M86.041, M86.042, M86.049, M86.141, M86.142, M86.149, M86.241, M86.242, M86.249, M86.051, M86.052, M86.059, M86.151, M86.152, M86.159, M86.251, M86.252, M86.259, M86.061, M86.062, M86.069, M86.161, M86.162, M86.169, M86.261, M86.262, M86.269, M86.071, M86.072, M86.079, M86.171, M86.172, M86.179, M86.271, M86.272, M86.279, M86.08, M86.18, M86.28, M86.09, M86.19, M86.29, M46.20, M46.21, M46.22, M46.23, M46.24, M46.25, M46.26, M46.27, M46.28, M86.9, M46.20, M46.21, M46.22, M46.23, M46.24, M46.25, M46.26, M46.27, M46.28 |  |
| Septic Arthritis | 711.0X  M00.00, M00.10, M00.20, M00.80, M00.9, M00.011, M00.012, M00.019, M00.111, M00.112, M00.119, M00.211, M00.212, M00.219, M00.811, M00.812, M00.819, M00.021, M00.022, M00.029, M00.121, M00.122, M00.129, M00.221, M00.222, M00.229, M00.821, M00.822, M00.829, M00.031, M00.032, M00.039, M00.131, M00.132, M00.139, M00.231, M00.232, M00.239, M00.831, M00.832, M00.839, M00.041, M00.042, M00.049, M00.141, M00.142, M00.149, M00.241, M00.242, M00.249, M00.841, M00.842, M00.849, M00.051, M00.052, M00.059, M00.151, M00.152, M00.159, M00.251, M00.252, M00.259, M00.851, M00.852, M00.859, M00.061, M00.062, M00.069, M00.161, M00.162, M00.169, M00.261, M00.262, M00.269, M00.861, M00.862, M00.869, M00.071, M00.072, M00.079, M00.171, M00.172, M00.179, M00.271, M00.272, M00.279, M00.871, M00.872, M00.879, M00.08, M00.18, M00.28, M00.88, M00.9, M00.09, M00.19, M00.29, M00.89 |  |
| Vascular device infection | 996.61,996.62  T82.6XXA, T82.7XXA |  |
| CIED (cardiac device-Pace maker, ICD) | V45.01, V45.02  Z95.0, Z95.810 | 33202-33226, 33227-33231, 33240, 33249, 33262-33264, 33270-33273 |
| Echocardiogram (TEE/TTE) |  | 93312-93318, 93355 (TEE)  93306-93308, 93350-93352 (TTE) |
| Cardiac valve replacement Surgery |  | 35.0x, 35.2x  33361-33366, 33405-33413, 33430, 33465, 33475  02RF37Z, 02RF38Z, 02RF3JZ, 02RF3KZ  X2RF332, 02RF37H, 02RF38H, 02RF3JH  02RF3KH, 02RH37Z, 02RH38Z, 02RH3JZ  02RH3KZ, 02RH37H, 02RH38H, 02RH3JH  02RH3KH, 02RF37Z, 02RF38Z, 02RF3JZ  02RF3KZ, 02RG37H, 02RG37Z, 02RG38H  02RG38Z, 02RG3JH, 02RG3JZ, 02RG3KH  02RG3KZ, 02RH37Z, 02RH38Z, 02RH3JZ  02RH3KZ, 02RJ37H, 02RJ37Z, 02RJ38H  02RJ38Z, 02RJ3JH, 02RJ3JZ,02RJ3KH  02RJ3KZ, 02RF07Z, 02RF08Z ,02RF0JZ  02RF0KZ, 02RF47Z ,02RF48Z, 02RF4JZ  02RF4KZ, 02RG07Z, 02RG08Z, 02RG0JZ  02RG0KZ, 02RG47Z, 02RG48Z, 02RG4JZ  02RG4KZ, 02RH07Z, 02RH08Z, 02RH0JZ  02RH0KZ, 02RH47Z, 02RH48Z, 02RH4JZ  02RH4KZ, 02RJ07Z, 02RJ08Z, 02RJ0JZ  02RJ0KZ, 02RJ47Z, 02RJ48Z, 02RJ4JZ  02RJ4KZ, 02RF07Z, 02RF08Z ,02RF0KZ  02RF47Z, 02RF48Z, 02RF4KZ, X2RF032  X2RF432, 02RF0JZ, 02RF4JZ, 02RG07Z  02RG08Z, 02RG0KZ, 02RG37Z, 02RG38Z  02RG3KZ, 02RG47Z, 02RG48Z, 02RG4KZ  02RG0JZ, 02RG3JZ, 02RG4JZ, 02RH07Z  02RH08Z, 02RH0KZ, 02RH47Z, 02RH48Z  02RH4KZ ,02RH0JZ, 02RH4JZ, 02RJ07Z  02RJ08Z, 02RJ0KZ, 02RJ37Z, 02RJ38Z  02RJ3KZ, 02RJ47Z, 02RJ48Z, 02RJ4KZ  02RJ0JZ, 02RJ3JZ, 02RJ4JZ |
| Pneumonia | 003.22, 020.3, 020.4, 020.5, 021.2, 022.1, 039.1, 073.0, 073.7, 073.8, 073.9, 083.0, 480.0, 480.1, 480.2, 480.3, 480.8, 480.9, 481, 482.0, 482.1, 482.2, 482.3, 482.30, 482.31, 482.32, 482.39, 482.4, 482.40, 482.41, 482.42, 482.49, 482.8, 482.81, 482.82, 482.83, 482.84, 482.89, 482.9, 483, 483.0, 483.1, 483.8, 484, 484.1, 484.3, 484.5, 484.6, 484.7, 484.8, 485, 486, 510.0, 510.9, 513.0, 997.31  A02.22, A20.2, A21.2, A22.1, A37.01, A37.11, A37.81, A37.91, A42.0, A43.0, A48.1, A70, A78, B25.0, B44.0, B77.81, J12.0, J12.1, J12.2, J12.3, J12.81, J12.89, J12.9, J13, J14, J15.0, J15.1, J15.20, J15.211, J15.212, J15.29, J15.3, J15.4, J15.5, J15.6, J15.7, J15.8, J15.9, J16.0, J16.8, J17, J18.0, J18.1, J18.8, J18.9, J85.0, J85.1, J85.2, J86.0, J86.9, J95.851 |  |
| Skin and Soft Tissue infection | 020.1, 021.0, 022.0, 032.85, 035, 039.0, 039.3, 039.4, 039.8, 039.9, 040.0, 040.1, 040.2, 040.3, 040.42, 040.81, 078.3, 082.0, 082.1, 082.2, 082.3, 082.40, 082.41, 082.49, 082.8, 082.9, 083.0, 083.1, 083.2, 083.8, 083.9, 087.0, 087.1, 087.9, 088.0, 088.81, 088.82, 088.89, 088.9, 098.50, 567.31, 680.0, 680.1, 680.2, 680.3, 680.4, 680.5, 680.6, 680.7, 680.8, 680.9, 681.00, 681.01, 681.02, 681.10, 681.11, 681.9, 682.0, 682.1, 682.2, 682.3, 682.4, 682.5, 682.6, 682.7, 682.8, 682.9, 683, 684, 685.0, 686.0, 686.00, 686.01, 686.09, 686.1, 686.8, 686.9, 675.00, 675.01, 675.02, 675.03, 675.04, 675.10, 675.11, 675.12, 675.13, 675.14, 675.80, 675.81, 675.82, 675.83, 675.84, 675.90, 675.91, 675.92, 675.93, 675.94, 705.83, 727.89, 7280, 728.86, 958.3, 997.62, 999.31, 999.33, 999.34, 999.39  A20.1, A21.0, A22.0, A28.1, A36.3, A42.2, A42.81, A42.82, A42.89, A42.9, A43.1, A43.8, A43.9, A44.0, A44.1, A44.8, A44.9, A46, A48.0, A48.52, A48.8, A54.42, A68.0, A68.1, A68.9, A69.20, A69.21, A69.22, A69.23, A69.29, A77.0, A77.1, A77.2, A77.3, A77.40, A77.41, A77.49, A77.8, A77.9, A78, A79.0, A79.1, A79.81, A79.89, A79.9, B47.0, B47.1, B47.9, B60.0, B60.8, B64, B78.1, E83.2, K12.2, K68.12, K90.81, L01.00, L01.01, L01.02, L01.03, L01.09, L01.1, L02.01, L02.02, L02.03, L02.11, L02.12, L02.13, L02.211, L02.212, L02.213, L02.214, L02.215, L02.216, L02.219, L02.221, L02.222, L02.223, L02.224, L02.225, L02.226, L02.229, L02.231, L02.232, L02.233, L02.234, L02.235, L02.236, L02.239, L02.31, L02.32, L02.33, L02.411, L02.412, L02.413, L02.414, L02.415, L02.416, L02.419, L02.421, L02.422, L02.423, L02.424, L02.425, L02.426, L02.429, L02.431, L02.432, L02.433, L02.434, L02.435, L02.436, L02.439, L02.511, L02.512, L02.519, L02.521, L02.522, L02.529, L02.531, L02.532, L02.539, L02.611, L02.612, L02.619, L02.621, L02.622, L02.629, L02.631, L02.632, L02.639, L02.811, L02.818, L02.821, L02.828, L02.831, L02.838, L02.91, L02.92, L02.93, L03.011, L03.012, L03.019, L03.021, L03.022, L03.029, L03.031, L03.032, L03.039, L03.041, L03.042, L03.049, L03.111, L03.112, L03.113, L03.114, L03.115, L03.116, L03.119, L03.121, L03.122, L03.123, L03.124, L03.125, L03.126, L03.129, L03.211, L03.212, L03.221, L03.222, L03.311, L03.312, L03.313, L03.314, L03.315, L03.316, L03.317, L03.319, L03.321, L03.322, L03.323, L03.324, L03.325, L03.326, L03.327, L03.329, L03.811, L03.818, L03.891, L03.898, L03.90, L03.91, L04.0, L04.1, L04.2, L04.3, L04.8, L04.9, L05.01, L05.02, L08.0, L08.1, L08.81, L08.82, L08.89, L08.9, L73.2, L88, L92.8, L98.0, L98.3, M60.000, M60.001, M60.002, M60.003, M60.004, M60.005, M60.009, M60.011, M60.012, M60.019, M60.021, M60.022, M60.029, M60.031, M60.032, M60.039, M60.041, M60.042, M60.043, M60.044, M60.045, M60.046, M60.051, M60.052, M60.059, M60.061, M60.062, M60.069, M60.070, M60.071, M60.072, M60.073, M60.074, M60.075, M60.076, M60.077, M60.078, M60.08, M60.09, M65.00, M65.011, M65.012, M65.019, M65.021, M65.022, M65.029, M65.031, M65.032, M65.039, M65.041, M65.042, M65.049, M65.051, M65.052, M65.059, M65.061, M65.062, M65.069, M65.071, M65.072, M65.079, M65.08, M67.20, M67.211, M67.212, M67.219, M67.221, M67.222, M67.229, M67.231, M67.232, M67.239, M67.241, M67.242, M67.249, M67.251, M67.252, M67.259, M67.261, M67.262, M67.269, M67.271, M67.272, M67.279, M67.28, M67.29, M67.80, M67.811, M67.812, M67.813, M67.814, M67.819, M67.821, M67.822, M67.823, M67.824, M67.829, M67.831, M67.832, M67.833, M67.834, M67.839, M67.841, M67.842, M67.843, M67.844, M67.849, M67.851, M67.852, M67.853, M67.854, M67.859, M67.861, M67.862, M67.863, M67.864, M67.869, M67.871, M67.872, M67.873, M67.874, M67.879, M67.88, M67.89, M71.00, M71.011, M71.012, M71.019, M71.021, M71.022, M71.029, M71.031, M71.032, M71.039, M71.041, M71.042, M71.049, M71.051, M71.052, M71.059, M71.061, M71.062, M71.069, M71.071, M71.072, M71.079, M71.08, M71.09, M71.80, M71.811, M71.812, M71.819, M71.821, M71.822, M71.829, M71.831, M71.832, M71.839, M71.841, M71.842, M71.849, M71.851, M71.852, M71.859, M71.861, M71.862, M71.869, M71.871, M71.872, M71.879, M71.88, M71.89, M72.6, N98.0, O91.011, O91.01, O91.013, O91.019, O91.02, O91.03, O91.111, O91.112, O91.113, O91.119, O91.12, O91.13, O91.211, O91.212, O91.213, O91.219, O91.22, O91.23, T798.XXA, T80.212A, T80.218A, T80.219A, T80.22XA, T80.29XA, T87.40, T87.41, T87.42, T87.43, T87.44, T88.0XXA |  |
| Prosthetic joint infection | 996.66, 996.67  T84.5, T84.50XA, T84.51XA, T84.52XA, T84.53XA, T84.54XA, T84.59XA, T84.60XA, T84.610A, T84.611A, T84.612A, T84.613A, T84.614A, T84.615A, T84.619A, T84.620A, T84.621A, T84.622A, T84.623A, T84.624A, T84.625A, T84.629A, T84.63XA, T84.69XA, T84.7XXA |  |
| Central line associated blood stream infection(CLABSI) | 999.32  T80.211A |  |
| Surgical Site Infections | 539.01, 539.81, 9985, 998.51, 998.59, 996.60, 996.61, 996.62, 996.63, 996.65, 996.66, 996.67, 996.68, 996.69  K68.11, K95.01, K95.81, T81.4XXA, T81.4XXD, T81.4XXS, T82.6XXA, T82.6XXD,T82.6XXS, T82.7XXA, T82.7XXD, T82.7XXS, T83.590A, T83.590D, T83.590S, T83.591A, T83.591D, T83.591S, T83.592A, T83.592D, T83.592S, T83.593A, T83.593D, T83.593S, T83.598A, T83.598D, T83.598S, T83.59XA, T83.59XD, T83.59XS, T83.61XA, T83.61XD, T83.61XS, T83.62XA, T83.62XD, T83.62XS, T83.69XA, T83.69XD, T83.69XS, T83.6XXA, T83.6XXD, T83.6XXS, T84.50XA, T84.50XD, T84.50XS, T84.51XA, T84.51XD, T84.51XS, T84.52XA, T84.52XD, T84.52XS, T84.53XA, T84.53XD, T84.53XS, T84.54XA, T84.54XD, T84.54XS, T84.59XA, T84.59XD, T84.59XS, T84.60XA, T84.60XD, T84.60XS, T84.610A, T84.610D, T84.610S, T84.611A, T84.611D, T84.611S, T84.612A, T84.612D, T84.612S, T84.613A, T84.613D, T84.613S, T84.614A, T84.614D, T84.614S, T84.615A, T84.615D, T84.615S, T84.619A, T84.619D, T84.619S, T84.620A, T84.620D, T84.620S, T84.621A, T84.621D, T84.621S, T84.622A, T84.622D, T84.622S, T84.623A, T84.623D, T84.623S, T84.624A, T84.624D, T84.624S, T84.625A, T84.625D, T84.625S, T84.629A, T84.629D, T84.629S, T84.63XA, T84.63XD, T84.63XS, T84.69XA, T84.69XD, T84.69XS, T84.7XXA, T84.7XXD, T84.7XXS, T85.71XA, T85.71XD, T85.71XS, T85.72XA, T85.72XD, T85.72XS, T85.730A, T85.730D, T85.730S, T85.731A, T85.731D, T85.731S, T85.732A, T85.732D, T85.732S, T85.733A, T85.733D, T85.733S, T85.734A, T85.734D, T85.734S, T85.735A, T85.735D, T85.735S, T85.738A, T85.738D, T85.738S, T85.79XA, T85.79XD, T85.79XS, T86.842 |  |

**Supplementary Table 2: Factors associated with readmission within 90 days Univariable analysis.**

| Variable  N (%) or Median (IQR) | Readmission with the same infection category | | | | | Any readmission within 90 days | | | |
| --- | --- | --- | --- | --- | --- | --- | --- | --- | --- |
|  | Yes  366 (19.3) | No  1529 (80.7) | | P value | OR [95% CI] | Yes  535 (28.3) | No  1360 (71.7) | P value | OR [95% CI] |
| **Age (years) mean** | 50.7 (18-64) | 50.8(18-64) | 0.471 | |  | 50.8(18-64) | 50.7 (18-64)) | 0.886 |  |
| **Age categories (years)**  **18-40** | 71 (19.4) | 261 (17.1) | 0.278 | | 1.45 [0.98-2.14] | 102(19.1)) | 230(16.7) | 0..275 | 1.36=3[0.97-1.89] |
| **41-50** | 60 (16.4) | 319 (20.9) | Ref | | Ref | 93(35.3) | 289(21.3) | Ref | Ref |
| **51-60** | 132 (36.1) | 575 (37.6) | 0.794 | | 1.25 [0.89-1.76] | 189(35.3) | 518(38.1)) | 0.268 | 1.18[0.82-1.48] |
| **61-64** | 103 (28.1) | 374 (24.5) | 0.030 | | 1.04[1.04-2.14] | 151(28.2) | 326(28.2) | 0.043 | 1.44[1.06-1.96] |
| **Sex (Males)** | 229 (62.6) | 963 (63.0) | 0.706 | | 0.95[0.75-1.27] | 332(62.1) | 860(63.2) | 0.516 | 0.93[0.75-1.15] |
| Residing in an urban area | 309 (87.5) | 1306 (87.7) | 0.96 | | 0.85[0.60-1.22] | 453(87.5) | 1162(87.7) | 0.212 | 0.95[0.70-1.29] |
| **Comorbidities** |  |  |  | |  |  |  |  |  |
| Diabetes | 130 (35.5) | 500 (32.7) | 0.267 | | 1.15[0.90-1.46] | 179(33.4) | 451(33.1) | 0.683 | 0.97[0.80-1.23] |
| Chronic kidney disease | 32 (8.7) | 100 (6.6) | 0.092 | | 1.43[0.94-2.17] | 44(8.2) | 88(6.7) | 0.117 | 1.35[0.92-1.96] |
| CHF | 57 (15.5) | 138(9.3) | 0.002 | | 1.87[1.34-2.62] | 77(14.9) | 118(8.7) | 0.001 | 1.79[1.31-2.41] |
| Metastatic solid cancer | 29 (7.9) | 107 (7.0) | 0.450 | | 1.18[0.76-1.81] | 54(10.1) | 82(6.0) | 0.001 | 1.81[1.26-2.59] |
| Hematological Malignancies | 17 (4.6) | 41 (2.7) | 0.072 | | 1.72[0.96-3.10] | 23 (4.3) | 35(2.6) | 0.0631 | 1.68[0.97-2.90] |
| Valvular heart disease | 45 (12.3) | 159 (10.4) | 0.277 | | 1.23[0.86-1.72] | 72(13.4) | 132(9.7) | 0.014 | 1.47[1.08-1.90] |
| Peripheral vascular disease | 32 (8.7) | 96 (6.3) | 0.075 | | 1.48[0.96-2.27] | 46(8.6) | 82(6.0) | 0.023 | 1.56[1.05-2.25] |
| Obesity | 113 (30.9) | 369 (24.1) | 0.007 | | 1.40[1.09-1.84] | 153(28.6) | 329(24.1) | 0.047 | 1.26[1.07-1.59 |
| Drug abuse | 30 (8.2) | 74 (4.8) | 0.077 | | 1.60[1.02-2.54] | 44(8.2) | 60(4.1) | 0.004 | 1.83[1.26-2.79] |
| Recent hospitalization in the preceding 30 days | 99 (27.1) | 257 (16.8) | <0.001 | | 1.88 [1.41-2.43] | 145(27.1)) | 211(15.5) | <0.001 | 2.07[155] |
| CIED implantation during the past year | 28 (7.7) | 61 (4.0) | 0.004 | | 1.97[1.23-3.16] | 39(7.3) | 50(3.7) | 0.001 | 2.06[1.30-3.18] |
| Valve replaced the year before admission | 3 (0.8) | 10 (0.7) | 0.751 | | 1.28[0.31-4.63] | 5 (0.9) | 8(0.6) | 0.404 | 1.60[0.52-4.92] |
| **Index Admission Characteristics** |  |  |  | |  |  |  |  |  |
| Length of hospital stay (days) | 8 (6-12) | 7 (5-11) | <0.001 | |  | 8(6-12) | 7 (5-11) | <0.001 |  |
| Intensive Care Unit stay | 245 (66.9) | 712 (46.6) | <0.001 | | 2.36 [1.81-3.03] | 355(66.4) | 602(44.2) | <0.001 | 2.49[2.06=3.086] |
| Infectious Diseases consultation | 244 (66.7) | 1064 (69.6) | 0.240 | | 0.87[0.67-1.12] | 357 (66.7) | 951(69.9) | 0.234 | 0.86[0.70-1.07] |
|  |  |  |  | |  |  |  |  |  |
| Echocardiography done | 257 (70.2) | 1049 (68.6) | 0.605 | | 1.07[0.76-1.39] | 377(70.5) | 929(68.1) | 0.316 | 1.10[0.81-1.37] |
| Valve replacement surgery | 6 (1.6) | 23 (1.5) | 0.837 | | 1.10[0.41-2.76] | 9(1.7) | 20(1.5) | 0.56 | 1.15[0.52-2.59] |
| **Type of infection** |  |  |  | |  |  |  |  |  |
| Osteomyelitis | 68 (18.6) | 267 (17.5) | 0.601 | | 1.89[0.81-1.46] | 82(15.3) | 253(18.6) | 0.877 | 1.03[0.79-1.96] |
| Septic arthritis | 50 (13.7) | 201 (13.2) | 0.690 | | 1.08[0.77-1.56] | 62(11.6) | 189(13.9) | 0.177 | 0.80[0.59-1.16] |
| Prosthetic joint infection | 43 (11.8) | 119 (7.8) | 0.017 | | 1.58[1.03-2.36] | 56(10.4) | 106(7.8) | 0.078 | 1.36[0.96-1.95] |
| Central line associated bacteremia | 48 (13.1) | 127 (8.3) | 0.001 | | 1.79 [1.28-2.52] | 76(14.2) | 99(7.2) | <0.001 | 2.27[1.460-3.14] |
| Infection of vascular device | 46 (12.6) | 146 (9.6) | 0.114 | | 1.34[0.93-1.96] | 69(12.9) | 123(9.0) | 0.010 | 1.49[1.08-2.09] |
| Skin & soft tissue infection | 127 (34.7) | 630 (41.2) | 0.024 | | 0.75[059-093] | 175(32.7) | 582(42.7) | 0.001 | 0.65[0.58-0.81] |
| Surgical Site Infections | 126 (34.4) | 432 (28.3) | 0.050 | | 1.14[0.99-1.69] | 173(32.3) | 385(28.3) | 0.183 | 1.18[0.95-1.49] |
| Epidural Abscess | 42 (11.5) | 158 (10.3) | 0.394 | | 1.16[0.81-1.61] | 50(9.3) | 150(11.3) | 0.423 | 0.85[0.61-1.24] |
| Endocarditis | 71 (19.4) | 205 (13.4) | 0.003 | | 1.57[1.16-2.13] | 100(18.6) | 176(12.9) | 0.002 | 1.55[1.18-2.04] |
| Pneumonia | 58 (15.9) | 298 (19.5) | 0.110 | | 0.77[0.56-1.05] | 82(15.3) | 274(20.1) | 0.016 | 0.71[0.54-0.94] |
| **OPAT Antibiotic** |  |  |  | |  |  |  |  |  |
| Oxacillin/Nafcillin/ Cefazolin | 288(78.7) | 1147(75.2) | Ref | | Ref | 421(78.7) | 1014(74.6) | Ref | Ref |
| Ceftriaxone | 78 (21.3) | 382 (25.0) | 0..174 | | 0.82[0.63-1.09] | 114 (21.3) | 346(25.4) | 0.076 | 0.79[0.62-1.03] |

**Supplementary Table 3: Standardized mean differences of variables between treatment groups in the unadjusted and weighted population**

| **Variable** | **Unadjusted** | **Adjusted (weighted)** |
| --- | --- | --- |
| Age | 0.0207 | 0.0070 |
| Patient location (urban) | 0.0599 | 0.0193 |
| Sex | 0.1321 | 0.0530 |
| CHF | 0.0611 | 0.0680 |
| Diabetes | 0.0429 | 0.0017 |
| Chronic kidney disease | 0.0328 | -0.0010 |
| Hematological malignancies | -0.0182 | -0.0039 |
| Valvular heart disease | -0.1108 | 0.0499 |
| Metastatic Cancer | -0.0226 | 0.0030 |
| Obesity | -0.0531 | -0.0065 |
| CIED implantation during the past year | -0.0360 | 0.0351 |
| Valve replaced during the past year | -0.1108 | 0.0239 |
| Length of index hospital stay;days, (IQR) | -0.2238 | -0.0627 |
| Intensive Care Unit stay | -0.0132 | -0.0011 |
| Infectious Diseases consultation during index admission | 0.1381 | 0.0084 |
| Echocardiography done | -0.2867 | -0.0047 |
| Hospitalization in the 30 days prior to the index admission | -0.0854 | 0.0242 |
| Valve replaced during index admission | 0.0281 | 0.0197 |
| OPAT duration | 0.0467 | 0.0813 |
| Osteomyelitis | -0.0697 | -0.0157 |
| Septic arthritis | -0.0684 | 0.0285 |
| Prosthetic joint infection | -0.0666 | -0.0112 |
| Central line associated bacteremia | -0.0451 | 0.0247 |
| Infection of vascular device | -0.1708 | 0.0241 |
| Skin & soft tissue infection | -0.0921 | -0.0185 |
| Surgical site infections | -0.0810 | 0.0132 |
| Epidural abscess | -0.1851 | 0.0216 |
| Endocarditis | -0.1708 | -0.0094 |
| Pneumonia | 0.1050 | 0.0012 |

Standardized mean difference (SMD) calculated as the difference in means or proportions of a variable divided by the pooled standard deviation of the variable. All standardized mean difference estimates compare patients with MSSA septicemia who received ceftriaxone versus oxacillin/nafcillin/cefazolin. Standardized mean differences <0.1 indicate no substantial difference in means or proportions between groups. In the inverse probability of treatment (IPT)-weighted population, all measured baseline characteristics were well balanced between treatment groups (standardized mean differences < 0.10).

**Supplementary Table 4: Outcomes of patients with MSSA BSI in subgroup with Endocarditis (n=276)**

| **Outcomes** | Total | Oxacillin/ Nafcillin/Cefazolin | Ceftriaxone | P value** |
| --- | --- | --- | --- | --- |
| **Endocarditis Subgroup** | **276** | **229** | **47** |  |
| 90 Day All-Cause readmission | 100 (36.6) | 85 (37.1) | 15 (31.9) | 0.499 |
| Readmitted with the same infection category | 71 (25.7) | 62 (27.1) | 9 (19.1) | 0.257 |

** Chi-square test
